# GPT is an effective tool for multilingual psychological text analysis

**DOI:** 10.1073/pnas.2308950121

**Published:** 2024-08-12

**Authors:** Steve Rathje, Dan-Mircea Mirea, Ilia Sucholutsky, Raja Marjieh, Claire E. Robertson, Jay J. Van Bavel

**Affiliations:** ^a^Department of Psychology, New York University, New York, NY 10003; ^b^Department of Psychology, Princeton University, Princeton, NJ 08540; ^c^Department of Computer Science, Princeton University, Princeton, NJ 08540; ^d^Center for Neural Science, New York University, New York, NY 10003; ^e^Department of Strategy and Management, Norwegian School of Economics, Bergen 5045, Norway

**Keywords:** AI, GPT, large language models, text analysis, machine learning

## Abstract

The social and behavioral sciences have been increasingly using automated text analysis to measure psychological constructs in text. We explore whether GPT, the large-language model (LLM) underlying the AI chatbot ChatGPT, can be used as a tool for automated psychological text analysis in several languages. Across 15 datasets (*n* = 47,925 manually annotated tweets and news headlines), we tested whether different versions of GPT (3.5 Turbo, 4, and 4 Turbo) can accurately detect psychological constructs (sentiment, discrete emotions, offensiveness, and moral foundations) across 12 languages. We found that GPT (*r* = 0.59 to 0.77) performed much better than English-language dictionary analysis (*r* = 0.20 to 0.30) at detecting psychological constructs as judged by manual annotators. GPT performed nearly as well as, and sometimes better than, several top-performing fine-tuned machine learning models. Moreover, GPT’s performance improved across successive versions of the model, particularly for lesser-spoken languages, and became less expensive. Overall, GPT may be superior to many existing methods of automated text analysis, since it achieves relatively high accuracy across many languages, requires no training data, and is easy to use with simple prompts (e.g., “is this text negative?”) and little coding experience. We provide sample code and a video tutorial for analyzing text with the GPT application programming interface. We argue that GPT and other LLMs help democratize automated text analysis by making advanced natural language processing capabilities more accessible, and may help facilitate more cross-linguistic research with understudied languages.

## Significance

Many fields—including psychology, sociology, communications, political science, and computer science—use computational methods to analyze text data. However, existing text analysis methods have a number of shortcomings. Dictionary methods, while easy to use, are often not very accurate when compared to recent methods. Machine learning models, while more accurate, can be difficult to train and use. We demonstrate that the large-language model GPT is capable of accurately detecting various psychological constructs (as judged by manual annotators) in text across 12 languages, using simple prompts and no additional training data. GPT thus overcomes the limitations present in existing methods. GPT is also effective in several lesser-spoken languages, which could facilitate text analysis research from understudied contexts.

Automated text analysis, or the analysis of written language through computational methods, is a rapidly growing tool for social and behavioral scientists (1–3). Because of the increasing availability of text data on the internet (e.g., social media sites and digitized book text), as well as the development of advanced machine learning methods, text analysis has been an increasingly useful tool for testing psychological questions with large datasets. The current paper examines whether text analysis can be made more effective and efficient by taking advantage of recent advances in AI.

The growing field of computational social science (4) has used automated text analysis for a variety of different purposes. For example, researchers have used text analysis tools to examine societal trends (5–8), explore what goes “viral” on social media (9–11), and identify linguistic correlates of mental health conditions (12, 13), ideology (14–16), and personality (17). Large text datasets are typically analyzed for the presence of various psychological constructs, such as sentiment (i.e., positivity versus negativity) (18–22), discrete emotions such as anger or sadness (23, 24), offensiveness (25), moral emotions (26, 27), out-party animosity (5, 9), or toxicity (28, 29).

Despite the promise and popularity of text analysis, existing text analysis methods have several major shortcomings. One popular text analysis method is dictionary analysis, which consists of counting the words of a certain category that are present in a text (e.g., counting the number of negative words in a tweet). This method is widely used within psychological research (9, 10, 18, 30–33). Several dictionary-based methods, such as the popular Linguistic Inquiry and Word Count (LIWC), have been psychometrically validated and correlate with an individual’s beliefs, behaviors, and psychological traits (34). However, dictionary methods are often not as accurate at detecting psychological constructs in text as determined by manual annotators—who are often considered the “gold standard” of accuracy in natural language processing (35)—when compared to more recent methods. This is in part because they do not consider the broader context of a sentence (1, 34). As such, there is a need for more accurate text analysis methods.

Machine learning methods have shown promise at accurately detecting psychological constructs in text data. For instance, researchers have used supervised machine learning classifiers to detect positive and negative sentiment (19, 20), moral outrage (27), incivility (28), out-party hate vs. in-party love (36), and discrete emotions (23, 37). Recently, researchers have also started using large-language models (LLMs), or neural networks with many parameters that are based on the transformer architecture and trained on massive amounts of text data (38) for psychological text analysis (39). However, most machine learning models are time-consuming and resource-intensive to create. Moreover, they often require high coding proficiency to design or implement and tens of thousands of manually annotated texts to train (27).

A further shortcoming of both of these approaches is that they are not well-equipped to analyze multilingual data. While several dictionaries have been translated into other languages (34), this translation process is costly and time-intensive, and sociocultural constructs captured in dictionaries developed for one language may not transfer to another language and culture (40). Similarly, traditional machine learning models tend to only work in the language the model was trained on. This makes it difficult to study the same constructs in multiple languages, which likely limits the generalizability of text analysis findings. Thus, like many other areas of the social and behavioral sciences which have been criticized for relying too heavily on Western, Educated, Industrialized, Rich, and Democratic (or WEIRD) populations and the English-language (41–44), text analysis may similarly be focusing on a narrow set of languages and cultures. As such, it is important to develop and validate language processing approaches that work across cultures.

We propose that GPT (45), the LLM developed by OpenAI that underlies the chatbot ChatGPT, has the potential to overcome the limitations present in both dictionary methods and machine learning methods for automated text analysis. GPT is trained on massive datasets of internet text (such as Common Crawl or Wikipedia), which makes it particularly promising for completing text analysis tasks across multiple languages without any additional training (known as “zero-shot” learning) (46). Compared to older LLMs like Bidirectional Encoder Representations from Transformers (BERT), newer versions of GPT (starting with 3.5) work by “prompting,” meaning that they generate output in response to a question asked by a human user. Thus, GPT can be asked the same questions as manual annotators (e.g., “how negative is this text on a scale of 1 to 7?”), making it more intuitive and flexible to use than traditional machine learning models. GPT has been lauded for its ability to exhibit human-level performance on a variety of tasks (e.g., passing the Bar Exam or acing the SAT test), and better performance than existing LLMs (47, 48). Researchers have also recently noted GPT’s ability to help with computational social science tasks (49–54), detect misinformation (55), infer politicians’ ideologies (56), write persuasive political arguments (57), respond to patient questions (58), simulate human research participants (59–61), and model collective behavior (56). Building on these findings, we examined GPT’s potential as a psychological text analysis tool across languages.

While other LLMs are effective for text analysis (38, 62–64), there is good reason to theorize that GPT might be superior in several ways. For instance, GPT has substantially more training data than prior language models and might work better for multilingual text analysis given its cross-linguistic data (45). GPT also has the benefit of being easy to use with simple prompts and little coding experience. Thus, GPT could provide a particularly powerful tool for a wide variety of scholars across the social and behavioral sciences (e.g., psychology, politics, sociology, communications, anthropology) who have limited experience in computational methods or who wish to conduct research outside English-speaking or Western samples.

## Overview

We tested the ability of three different versions of GPT (3.5 Turbo, GPT-4, and GPT-4 Turbo—an updated version of GPT-4 released in January 2024 and with training data up to December 2023) to accurately detect psychological constructs in text as judged by manual annotators across 15 datasets (*n* = 47,912 annotated tweets, news headlines, and Reddit comments, Table 1). Each of these datasets were manually annotated by human raters for the presence or absence of specific psychological constructs—sentiment, discrete emotions, offensiveness, and moral foundations. For each psychological construct, we first examined GPT’s performance in English as well as a second language from a different language family (Arabic, Indonesian, or Turkish) using six publicly available datasets with categorical labels (datasets 1 to 6). Then, we analyzed a dataset of news headlines rated for sentiment and discrete emotions on a Likert scale to examine how GPT performs with psychological scale ratings (65), a different type of text, and a dataset that was not publicly available on the internet and therefore could not have been used to train GPT (dataset 7).

**Table 1.** Description of datasets used

Dataset	Construct	Text type	Size of dataset	Labels	Language	Number of Speakers (millions)
Sentiment of English tweets (2017)	Sentiment	Tweets	12,283	Positive, Negative, Neutral	English	1,450
Sentiment of Arabic tweets (2017)	Sentiment	Tweets	6,100	Positive, Negative, Neutral	Arabic	630
Discrete emotions in English tweets (2020)	Discrete Emotions	Tweets	1,421	Anger, Joy, Sadness, Optimism	English	1,450
Discrete emotions in Indonesian tweets (2020)	Discrete Emotions	Tweets	440	Anger, Fear, Sadness, Love, Joy	Indonesian	300
Offensiveness in English tweets (2019)	Offensiveness	Tweets	860	Offensive, Not Offensive	English	1,450
Offensiveness in Turkish tweets (2020)	Offensiveness	Tweets	3,528	Offensive, Not Offensive	Turkish	88
Sentiment & discrete emotions in news headlines (2023)	Sentiment, Discrete emotions	News headlines	213	1 = very negative; 7 = very positive	English	1,450
Sentiment of African tweets (2023)	Sentiment	Tweets	748	Positive, Negative, Neutral	Swahili	220
	Sentiment	Tweets	1,000	Positive, Negative, Neutral	Hausa	72
	Sentiment	Tweets	1,000	Positive, Negative, Neutral	Amharic	57.5
	Sentiment	Tweets	1,000	Positive, Negative, Neutral	Yoruba	55
	Sentiment	Tweets	1,000	Positive, Negative, Neutral	Igbo	42
	Sentiment	Tweets	949	Positive, Negative, Neutral	Twi	17.5
	Sentiment	Tweets	1,026	Positive, Negative, Neutral	Kinyarwanda	15
	Sentiment	Tweets	234	Positive, Negative, Neutral	Tsonga	7
Moral Foundations in Reddit Comments (2022)	Moral Foundations	Reddit Comments	16,123	Care, Proportionality, Equality, Loyalty, Authority, Purity, Moral Sentiment	English	1,450

We used 15 different datasets which contained 47,925 manually annotated tweets and news headlines in 12 languages from various language families, annotated for four different psychological constructs (sentiment, discrete emotions, offensiveness, and moral foundations). Datasets 7 to 16 were not publicly available on the internet at the time GPT was trained in 2021, and thus could not have influenced the training dataset.

To examine whether GPT performed equally well with less commonly spoken or studied languages, we tested GPT’s ability to detect sentiment in eight African languages, such as Swahili, Amharic, Yoruba, and Kinyarwanda (datasets 8 to 15). Finally, we analyzed GPT’s ability to detect moral foundations—a more complex construct (dataset 16). For each dataset, we compared the performance of GPT to other common methods of text analysis, such as dictionary methods. We also compared the results of GPT to the top-performing fine-tuned machine learning models found in the papers associated with the datasets we analyzed.

## Results

For each of the 15 datasets (see Table 1 for descriptions), we used the GPT application programming interface (API) to repeatedly prompt GPT using R or Python code. We used simple prompts, such as “Is the sentiment of this text positive, neutral, or negative? Answer only with a number: 1 if positive, 2 if neutral, and 3 if negative. Here is the text: [tweet, news headline or Reddit comment text]” (see Table 2 for prompt summary). In most cases, we kept the GPT prompts as close as possible to the instructions that human annotators were provided (see *Methods* for details). Then, we examined how GPT’s performance aligned with human annotations, following the tradition in natural language processing of using human manual annotations as the gold standard (35).

**Table 2.** Prompt table

Sentiment analysis (categorical)	Emotion detection (categorical)	Offensiveness	Sentiment analysis (Likert)	Emotion detection (Likert)	Moral foundations
Is the sentiment of this (Arabic/Swahili/…) text positive, neutral, or negative? Answer only with a number: 1 if positive, 2 if neutral, and 3 if negative. Here is the text: *[Tweet text]*	Which of these [number of] emotions–[list of emotions]–best represents the mental state of the person writing the following (Indonesian) text? Answer only with a number: 1 if [emotion1], 2 if [emotion2], [...]. Here is the text: *[Tweet text]*	Is the following (Turkish) post offensive? Answer only with a number: 1 if offensive, and 0 if not offensive. Here is the post: *[Tweet text]*	How negative or positive is this headline on a 1 to 7 scale? Answer only with a number, with 1 being “very negative” and 7 being “very positive.” Here is the headline: *[Headline text]*	How much [emotion] is present in this headline on a 1 to 7 scale? Answer only with a number, with 1 being “no [emotion]” and 7 being “a great deal of [emotion].” Here is the headline: *[Headline text]*	Does the following Reddit comment express the moral foundation of [ moral foundation] (i.e., [definition of moral foundation])? Please answer only with a number: 1 if yes and 0 if no. Here is the Reddit comment: [*Reddit comment text]*

Shown are all the prompts used for each construct. Non-English prompts were derived from the English prompts by specifying the language the text was written in. Prompts in combination with the tweet or headline text were run for each text entry in the dataset using the GPT API.

We used two metrics that are traditionally used to measure the performance of machine learning models: accuracy and average *F1*. Accuracy is the number of correct ratings (i.e., the number of GPT outputs that matched the manual annotations) over the total number of ratings. Average *F1* is a more complex metric that takes into account the various types of errors made by GPT (false positives and false negatives) and is used frequently in the machine learning literature. See *Methods* for a detailed description of these performance metrics and see our OSF for code and datasets (https://osf.io/6pnb2/) (66). We also examined whether we could improve GPT’s accuracy by providing it with a few examples (known as “few-shot learning”) and comparing the results to those without any examples (“zero-shot learning”). Finally, we examined the test–retest reliability of GPT.

### Sentiment.

We first examined GPT’s ability to detect sentiment—or the overall positivity, negativity, or emotional neutrality expressed in text. To assess GPT’s performance across languages, we used manually annotated datasets of tweets in both English and Arabic (67). Both datasets came from the 2017 iteration of SemEval, a competition for designing machine learning methods for text analysis (*Methods*). Even the oldest GPT model we analyzed, GPT-3.5 Turbo, achieved good performance at predicting human ratings in both English (Accuracy = 0.673, *F1* = 0.685) and Arabic (Accuracy = 0.700, *F1* = 0.720) (Table 3). Moreover, GPT outperformed the best model from the SemEval competition in both languages (Table 4). This is not entirely surprising given that the original study is from 2017 and the models used were not large language models. Overall, GPT appears to be effective at multilingual sentiment analysis, with performance comparable to top-performing machine learning models from several years ago.

**Table 3.** GPT-3.5 Turbo, GPT-4, and GPT-4 Turbo Results

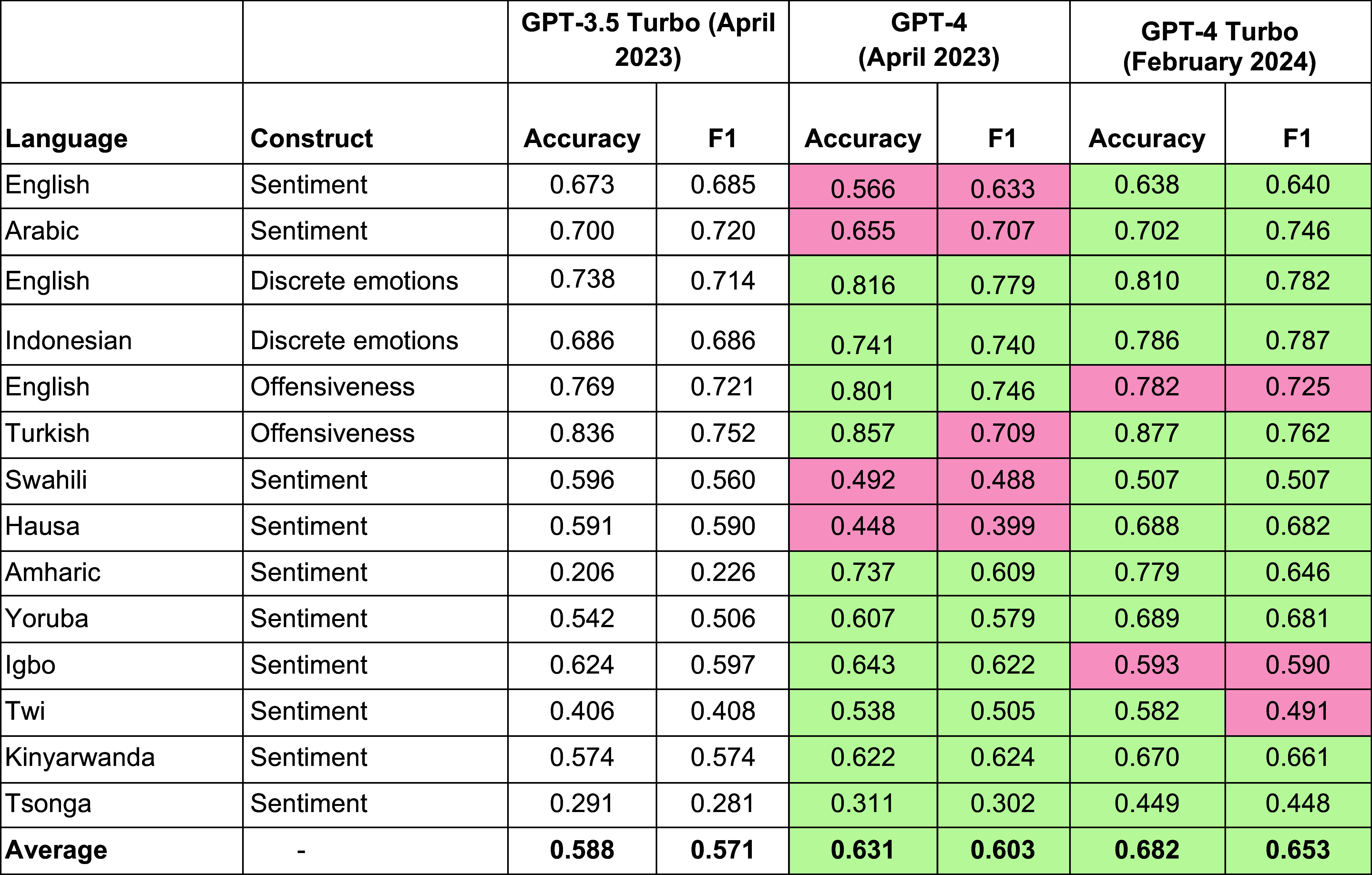

We report the ability of GPT-3.5 Turbo, GPT-4, and GPT-4 Turbo (released in January 2024) to accurately detect three psychological constructs (sentiment, discrete emotions, and offensiveness) across 12 languages. The average performance across languages and constructs improved with each iteration of GPT (with GPT-4 Turbo outperforming earlier versions). We report two performance metrics commonly used in machine learning: accuracy (number of correct ratings over total number of ratings), and *F1*, a more complex measurement that takes into account different types of classification errors (see *Methods* for a detailed description of performance metrics). Green indicates instances where a version of GPT was better than the previous version, and red indicates where a version of GPT was worse than the previous version. Precision and recall values for all datasets are given in *SI Appendix*, Table S1. These are zero-shot results—a comparison with few-shot results for GPT-4 (more detailed prompts that provide examples) can be found in *SI Appendix*, Table S2, and the prompts used for few-shot classification can be found in *SI Appendix*, Table S3.

**Table 4.** GPT-4 vs. top-performing machine learning models

Language	Construct	Top-performing GPT model F1	Top-performing GPT model	Top-performing alternate model F1	Model type	Year of study
English	Sentiment	0.685	3.5 Turbo	0.677	LSTM-CNN	2017
Arabic	Sentiment	0.746	4 Turbo	0.610	Naive Bayes	2017
English	Discrete emotions	0.782	4 Turbo	0.785	BERT	2020
Indonesian	Discrete emotions	0.785	4 Turbo	0.795		2020
English	Offensiveness	0.746	4	0.829		2019
Turkish	Offensiveness	0.762	4 Turbo	0.826	XLM-BERT	2020
Swahili	Sentiment	0.560	3.5 Turbo	0.657	Fine-tuned XLM-R	2023
Hausa	Sentiment	0.682	4 Turbo	0.826		
Amharic	Sentiment	0.646	4 Turbo	0.640		
Yoruba	Sentiment	0.681	4 Turbo	0.800		
Igbo	Sentiment	0.622	4	0.830		
Twi	Sentiment	0.505	4	0.675		
Kinyarwanda	Sentiment	0.661	4 Turbo	0.726		
Tsonga	Sentiment	0.448	4 Turbo	0.607		
Average	–	0.665	–	0.735	–	–

We compare the performance of GPT-3.5 and GPT-4 to the performance of the top machine learning models reported in the papers from which we retrieved the tested datasets. All top-performing model statistics (besides the GPT statistics) are taken from the papers from which the datasets originated. GPT sometimes outperformed the top-performing fine-tuned models, or at least came close to the performance of these top-performing models. The abbreviations are as follows: LSTM, Long Short Term Memory; CNN, Convolutional Neural Network; BERT, Bidirectional Encoder Representations from Transformers; XLM, Cross-Lingual Model; XLM-R, XLM combined with RoBERTa (a variant of BERT with more extensive pretraining).

Interestingly, GPT-3.5 Turbo performed slightly better than both GPT-4 and GPT-4 Turbo—both newer models—on both tasks (English: GPT-3.5 Turbo *F1* = 0.685, GPT-4 *F1*= 0.633, GPT-4 Turbo *F1* = 0.615; Arabic: GPT-3.5 Turbo *F1* = 0.720; GPT-4 *F1* = 0.707; GPT-4 Turbo *F1* = 0.690). Examination of the confusion matrices (*SI Appendix*, Fig. S1) revealed a possible driver of this effect: GPT-4 was more likely to classify “neutral” tweets as either “positive” or “negative” compared to GPT-3.5 Turbo in both English and Arabic, a bias which persisted in GPT-4 Turbo albeit to a lesser extent. This suggests more recent versions of GPT might have a cross-linguistic bias toward overestimating sentiment in a given text compared to humans.

### Discrete Emotions.

Next, we examined GPT’s ability to accurately detect more complex discrete emotions, such as anger, joy, fear, and sadness. To assess the GPT’s multilingual performance, and to see whether results generalize beyond English and Arabic, we compared English with another lesser-studied language from a completely different language family—Indonesian, once again using two existing datasets. We found that all versions of GPT had high agreement with humans in both English (GPT-3.5 Turbo *F1* = 0.720, GPT-4 *F1* = 0.779, GPT-4 Turbo *F1* = 0.782) and Indonesian (GPT-3.5 Turbo *F1* = 0.678, GPT-4 *F1* = 0.740, GPT-4 Turbo *F1* = 0.785) (Table 3). Each newer version of GPT showed an improvement in accuracy and F1 above the previous one, with GPT-4 Turbo reaching an F1 score that was roughly equivalent to the top-performing state-of-the-art LLM (a BERT model that was fine-tuned on Twitter data) in both English and Indonesian (Table 4). Full confusion matrices can be found in *SI Appendix*, Fig. S2.

### Offensiveness.

We then examined GPT’s ability to detect a different psychological construct, offensiveness, in both English and Turkish (25, 68). Offensive text was defined as text that “includes insults, threats, and posts containing any form of untargeted profanity” (25). We found high agreement between all versions of GPT and human ratings for English (*F1* = 0.725 to 0.746) and Turkish (*F1* = 0.709 to 0.762). However, the performance did not reach that of the top-performing models from their respective studies, both of which used older LLMs that were fine-tuned with additional training data (*F1* = 0.826 to 0.829; Table 4). The different GPT versions had similar confusion matrices, although more recent versions were more likely to label Turkish tweets as “not offensive” (*SI Appendix*, Fig. S3).

### Sentiment and Discrete Emotions Measured on a Continuous Scale.

GPT is capable of accurately detecting psychological constructs in text, with performance comparable to several top-performing, fine-tuned machine learning models. However, it is unclear whether this performance generalizes to other types of text data besides Tweets. Moreover, it is unclear whether GPT performs similarly with other types of ratings, such as Likert scales (e.g., 1 = strongly disagree to 7 = strongly agree), which are commonly used in psychology and the social sciences. Finally, since all of the datasets used so far were publicly available on the internet, it is possible that they were part of GPT’s training set.

**Fig. 1.**
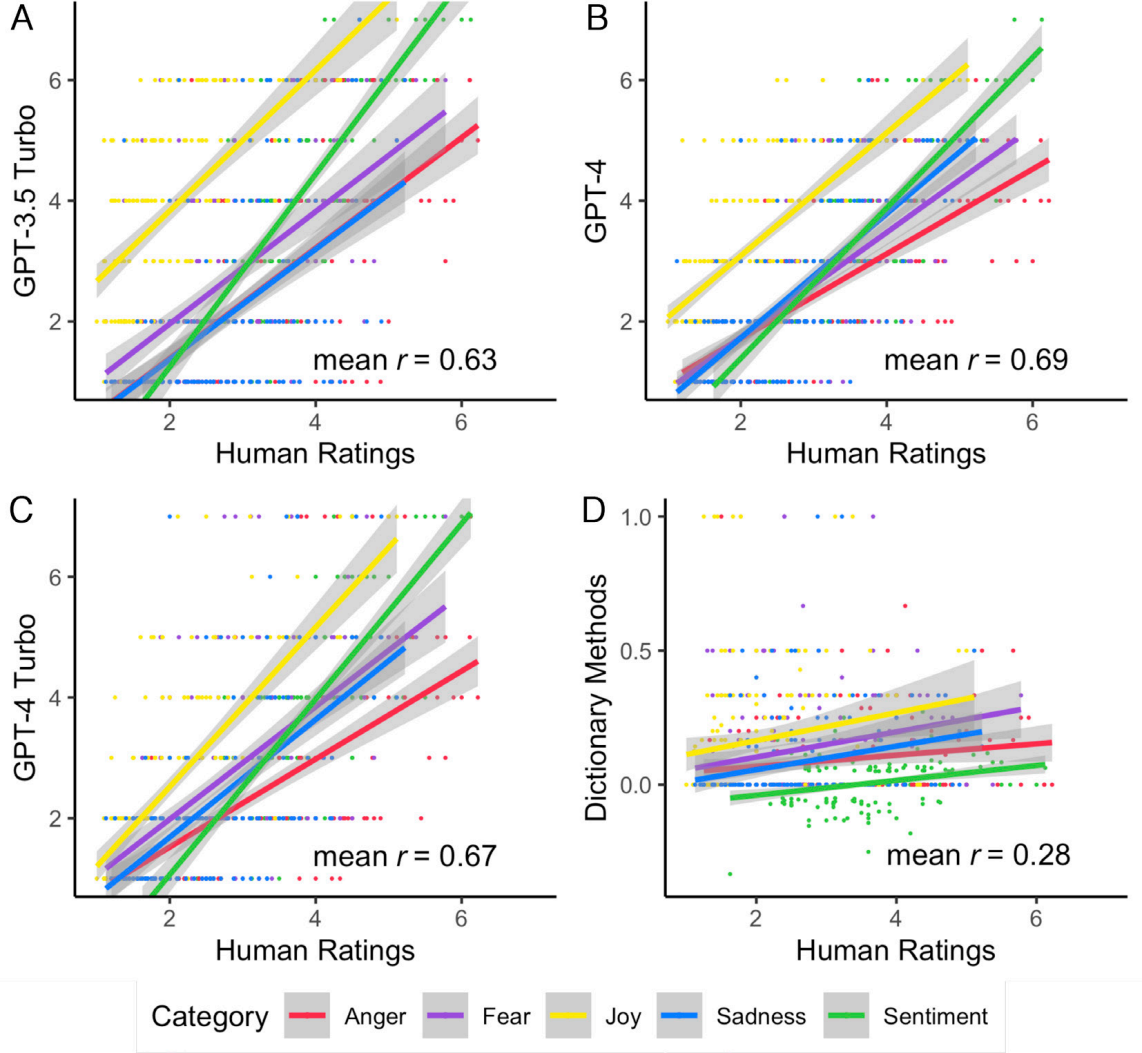
Scatterplots showing correlations between human ratings and ratings predicted by different text analysis methods. (*A*) GPT-3.5 ratings; (*B*) GPT-4 ratings; (*C*) GPT-4 Turbo Ratings (the most recent model as of February 2024), and (*D*) ratings computed using dictionary methods (LIWC and NRC dictionaries with negation handling). Data are from 213 manually annotated headlines (measured on a Likert scale from 1 to 7). Each line represents a separate correlation between GPT output and manual annotators for a separate construct.

To address these considerations, we analyzed a recent dataset of news headlines annotated for sentiment and four discrete emotions using 1 to 7 Likert scales (18). This dataset was accessed upon request from the study authors, meaning it was likely not a part of GPT’s training dataset.^*^ The prompts for Likert scales were slightly different (e.g., “How negative or positive is this headline on a 1 to 7 scale?”; see Table 2 for prompts). We found very high correlations (*r* = 0.56 to 0.74) between GPT-3.5 Turbo and human ratings, and even higher correlations for GPT-4 (*r* = 0.66 to 0.75) and GPT-4 Turbo (*r* = 0.59 to 0.77) (Fig. 1 and Table 5). This suggests that GPT is capable of accurately detecting psychological constructs in text, regardless of the format of the ratings or the type of text.

**Table 5.** GPT vs. dictionary methods (LIWC and NRC discrete emotions)

Psychological construct	Spearman correlation with manual annotators’ ratings				Spearman correlation between GPT-3.5 Turbo and GPT-4 output	Spearman correlation between GPT-3.5 Turbo and GPT-4 Turbo output
Method	GPT-3.5 Turbo	GPT-4	GPT-4 Turbo	Dictionary methods		
Sentiment	0.74	0.75	0.77	0.30	0.79	0.86
Anger	0.64	0.69	0.64	0.22	0.72	0.80
Fear	0.56	0.66	0.59	0.29	0.72	0.79
Joy	0.64	0.70	0.70	0.30	0.79	0.82
Sadness	0.56	0.67	0.64	0.30	0.67	0.76
Average	0.63	0.69	0.67	0.28	0.74	0.80

We show the Spearman correlation between the ratings from GPT-3.5 and GPT-4 and the ratings of manual annotators for sentiment and discrete emotions. We compare this to the correlation between dictionary methods (LIWC and NRC dictionaries with negation handling) and the ratings of manual annotators. Data are from 213 manually annotated headlines (measured on a Likert scale from 1 to 7) taken from ref. 18.

We also compared GPT’s performance to the performance of two popular dictionary methods used in the study the dataset was taken from: the LIWC method of measuring sentiment (34), and the National Research Council Canada (NRC) Emotion Lexicon (69) method of measuring discrete emotions. Dictionary scores also included negation handling. Specifically, in the original dictionary analyses, any emotional word that appeared within three words after a negation word was reverse coded. Thus, phrases like “not happy,” “not too happy,” or “not looking too good” were all coded as negative. We use the dictionary scores calculated from the original author, who describes how they calculated these scores in detail in the original paper (17). The correlations between these dictionary-based methods and manual annotations were much smaller (between *r* = 0.22 and *r* = 0.30) than the correlations between manual annotations and both versions of GPT. Z-tests found that all of the correlations between manual annotations and GPT output were significantly different from the correlations between manual annotations and dictionary methods (all *Ps* < 0.001). Thus, GPT appears to be far more effective at detecting manually annotated sentiment and discrete emotions than common dictionary-based methods that are very popular in psychology and the social sciences.

One potential limitation of using GPT is that it continues to evolve over time and may provide very different estimates from one version to the next. To address this possibility, we tested the Spearman correlation between the outputs of successive versions of GPT on this dataset (Table 5). We found very high correlations between GPT-3.5 Turbo and GPT-4 (between *r* = 0.67 and *r* = 0.79), as well as high correlations between GPT-4 and GPT-4 Turbo (between *r* = 0.76 and *r* = 0.86), indicating that different versions of GPT provide very similar (albeit not exact) output for text analysis problems.

We also ran correlations between GPT-3.5 Turbo, GPT-4, and GPT-4 Turbo and the dictionary method output (*SI Appendix*, Table S4). We found positive correlations ranging from between 0.12 and 0.38 when looking at the relationship between dictionary methods and GPT output. Thus, while GPT output and dictionary method output were correlated, the correlations were not particularly strong, suggesting that analyses using GPT may potentially lead to very different conclusions than analyses using dictionary methods.

### Sentiment in Lesser-Spoken African Languages.

Our analyses so far have focused on languages that are widely spoken and thus are highly represented in GPT’s training data. To see whether GPT is effective for languages that are less likely to be represented in the training data, we took advantage of a recent collection of tweets manually annotated for sentiment in multiple African languages (70). We chose eight of these languages—three of which had less than 20 million speakers (Table 1).

Overall, GPT was effective even with lesser-spoken African languages (Table 3). Further, GPT’s average performance at detecting sentiment with African languages improved dramatically from GPT-3.5 (Mean Accuracy = 0.462; Mean *F1* = 0.455) to GPT-4 (Mean Accuracy = 0.558, Mean *F1* = 0.520) to GPT-4 Turbo (Mean Accuracy = 0.636, Mean *F1* = 0.600). Initially, GPT had less-than-chance performance on two languages: Tsonga (GPT-3.5 Turbo Accuracy = 0.291; *F1* = 0.281), the least-spoken language we analyzed, and Amharic (GPT-3.5 Turbo Accuracy = 0.646; *F1* = 0.226). However, GPT’s performance on these languages improved considerably with the release of GPT-4 (Tsonga *F1* = 0.302; Amharic *F1* = 0.609) and GPT-4 Turbo (Tsonga *F1* = 0.448, Amharic *F1* = 0.646). These results suggest that GPT is effective at detecting psychological constructs even in lesser-spoken languages. The recent performance improvements also suggest that GPT is rapidly improving with newer models.

Despite GPT’s relatively high-performance and the improvement across versions, GPT generally lags behind state-of-the-art fine-tuned models. Specifically, the average performance of the top-performing GPT version for each African language (Mean *F1* = 0.600) was lower than the average performance for all fine-tuned large language models (Mean *F1* = 0.720). However, the top-performing model was a fine-tuned, cross-lingual LLM (Afro-XLMR) that was trained on a subset of Tweets from the same dataset. Given that GPT had no fine-tuning on manually annotated datasets, this lower performance is not entirely surprising. For one language (Amharic), GPT-4 Turbo (*F1* = 0.646) outperformed the top-performing fine-tuned model (*F1* = 0.640).

### Moral Foundations.

We also conducted supplementary analyses (*SI Appendix*, Tables S5–S8) testing GPT-4 and GPT-4 Turbo on a more complex set of constructs: moral foundations. Specifically, we examined a dataset of 16,123 Reddit comments that were manually annotated for six specific moral foundations (i.e., care, proportionality, equality, purity, authority, loyalty). These moral foundations were derived from work in Moral Foundations Theory, which states that people base their moral judgments on a few core foundations (71). The dataset also had comments annotated for “thin morality” (72) and overall moral sentiment (i.e., whether the text contains at least one of the six foundations or thin morality). We measure GPT’s ability to detect the six foundations as well as moral sentiment.

Although each moral foundation had a very high accuracy score (ranging from 0.899 to 0.980 for each foundation, and 0.634 to 0.684 for moral sentiment overall), this was mainly driven by true negatives (i.e., comments that did not contain a moral foundation that were correctly labeled by GPT), as there much fewer positives (i.e., comments that contained a moral foundation). As a result, the average *F1*, precision, and recall scores—which balance the accurate detection of positives and negatives—were considerably lower. While *F1* scores were relatively high for care (GPT-4 *F1* = 0.534, GPT-4 Turbo *F1* = 0.497) and moral sentiment (GPT-4 *F1* = 0.653, GPT-4 Turbo *F1 =* 0.677), they were lower for foundations such as proportionality (*GPT-4 F1* = 0.174, GPT-4 Turbo *=* 0.130) and purity (*GPT-4 F1* = 0.192, GPT-4 Turbo *F1* = 0.144). Thus, GPT may struggle more with more complex or difficult-to-define constructs.

When compared to a fine-tuned BERT model, GPT performed worse, although it came close to the fine-tuned BERT model for moral sentiment (*SI Appendix*, Tables S5 and S6). However, GPT outperformed a fine-tuned BERT model that was trained on Twitter data and applied to Reddit data for several moral foundations (*SI Appendix*, Tables S7 and S8). This suggests that fine-tuned models, while highly accurate for one context or dataset, are not very flexible when applied to other contexts or datasets. What GPT loses in accuracy (compared to fine-tuned models) it gains in its flexibility, since it is highly accurate without any further training data on a variety of datasets. Additionally, GPT itself can be fine-tuned to further increase its performance. For instance, one paper found that fine-tuned GPT outperformed even fine-tuned BERT at detecting moral foundations (73).

### Test–Retest Reliability of GPT.

Even when the temperature is set to 0 (which provides GPT’s most deterministic output), GPT is not completely deterministic, and the responses of the same GPT model can vary (74). This could cause reproducibility issues when using GPT for psychological text analysis. To assess the test–retest reliability of GPT, we compared two different runs (1 d apart) of the most recent version of GPT (GPT-4 Turbo) on the eight African sentiment datasets. We found that the weighted Cohen’s kappa values ranged between 0.93 for Tsonga to 0.99 in Swahili, Hausa, Yoruba, and Kinyarwanda (*SI Appendix*, Table S9). A Cohen’s Kappa value of 0.81 indicates “almost perfect agreement,” indicating that running GPT at separate times yields extremely high reliability when compared to traditional standards (75).

Another question is whether the language in which the prompt is asked changes the results substantially. To test this, we compared two different runs of GPT-4 Turbo on the Indonesian discrete emotion dataset. For the second run, we translated the prompt into Indonesian using Google Translate. We found that the weighted Cohen’s kappa value between the English-prompted and the Indonesian-prompted runs was 0.95 (*SI Appendix*, Table S7), once again indicating almost perfect agreement. This suggests that GPT provides extremely reliable results even when the prompt is asked in a different language.

## Discussion

We tested whether recent advances in AI—specifically, the popular large language model GPT—could help make automated text analysis more effective and efficient. Across 16 different datasets, we found that multiple versions of GPT (GPT-3.5, GPT-4, and GPT-4 Turbo) could accurately detect various psychological constructs (sentiment, discrete emotions, and offensiveness, and moral foundations) in different types of text (tweets, news headlines, and Reddit posts) and across 12 languages, including lesser-spoken African languages (76). GPT performs much better than English-language dictionary methods at both sentiment analysis and discrete emotion detection. In many cases, GPT performed close to (and sometimes better than) fine-tuned machine learning models. However, the performance of GPT was often lower than the performance of more recent fine-tuned models based on LLMs. GPT’s performance improved substantially from GPT-3.5 (Average *F1* = 0.571) to GPT-4 (Average *F1* = 0.603) to GPT-4 Turbo (Average *F1* = 0.653), with largest improvements for the least-spoken languages. These results suggest that GPT is an effective multilingual text analysis tool.

GPT may be superior to many—but not all—existing automated text analysis methods. While dictionary-based text analysis methods are often used because of their user-friendliness, GPT is also very easy to use and achieves much higher accuracy at detecting psychological constructs as judged by manual annotators. In some cases, GPT may also be a better choice than fine-tuned machine learning models. While machine learning classifiers require large amounts of manually annotated text to train and high coding proficiency, GPT does not require training data, is effective across contexts and languages, and is intuitive to use with little coding experience, since it works via prompting with minimal programming. We provide sample code for analyzing text data with GPT on our OSF: https://osf.io/6pnb2/ (66). We also provide a YouTube tutorial that demonstrates how to use GPT for text analysis in the R programming language: www.youtube.com/watch?v=Mm3uoK4Fogc&t=344.

Given its high-performance across languages, GPT could also facilitate more complex cross-linguistic and cross-cultural research that takes into account languages that are less commonly studied (and therefore lack existing dictionaries or fine-tuned models). This might help solve the issue of text analysis—and social science more broadly—focusing too much on WEIRD populations and English-language datasets. While GPT’s performance was initially worse than chance for some lesser-spoken and lesser-studied languages (such as Tsonga, which has 7 million global speakers), GPT-4 and GPT-Turbo showed major improvements for these languages. These improvements provide hope that GPT and other LLMs will continue to get better at text analysis tasks for lesser-studied languages, particularly as models become larger and incorporate more training data. Future research should continue to explore the accuracy of GPT and other LLMs across different languages and cultures to assess whether these findings generalize to other linguistic and cultural contexts that we did not measure.

We also explored the test–retest reliability of GPT, or the agreement between different runs of GPT on the same dataset. We found that reliability was very high (Cohen’s kappa = 0.93 to 0.99) if the same version of GPT was run multiple times. Note that our runs were only 1-d apart; other work has reported lower reliability if GPT runs are several months apart (74). This is potentially an issue for doing reproducible analysis; however, the output of human annotators, like GPT, is also not reproducible and usually has far lower test–retest reliability (73).

Finally, we experimented with providing GPT-4 with examples (“few-shot” learning) in an attempt to improve its performance, finding this sometimes did increase, but other times decreased performance (See *SI Appendix*, Tables S2 and S3 for examples of few-shot prompts). We encourage researchers to experiment with different GPT versions, prompts, and few-shot learning strategies for whatever construct they are measuring.

While we make the case that GPT (and other prompt-based large language models) might be better than several other text analysis tools due to its ease of use and high accuracy, there are several cases when researchers may want to consider existing methods. While we show that GPT surpasses the accuracy of dictionary methods at detecting manually annotated sentiment, researchers may still wish to use dictionary methods because the results are more interpretable or build on existing research. GPT is a “black box,” and it is difficult to know why it is producing the responses it provides. Thus, while GPT is good at predicting manually annotated sentiment with high accuracy, other more interpretable methods may also be useful for understanding psychological processes.

Additionally, while GPT works well without any fine-tuning (zero-shot), in most cases it did not surpass the accuracy of fine-tuned LLMs. Researchers may want to further fine-tune GPT (or other models like BERT), especially when working with more complex constructs, since we found that GPT struggled with such constructs (e.g., the moral foundation of purity, *SI Appendix*, Figs. S5–S8). While fine-tuned LLMs will often be more accurate than zero-shot GPT, a fine-tuned classifier trained on one dataset will not work as well when applied to a different kind of dataset. For instance, a classifier trained on Twitter data did not work as well when applied to Reddit data (*SI Appendix*, Tables S7 and S8). Thus, a major strength of GPT over fine-tuned classifiers is its flexibility across contexts.

We encourage those who use GPT for text analysis to be aware of its potential biases. Some work has found that LLMs reflect human biases, such as in-group favoritism (77). Other work has found that GPT is biased toward responses on cognitive tasks that are similar to those of WEIRD populations (78). These biases have led critics to warn against the thoughtless use of GPT as a tool to simulate human participants (79) or replace other forms of text analysis without consideration of these potential biases (73).

Despite GPT’s potential cultural biases, we still found that GPT was remarkably accurate at detecting the aggregated judgments of native speakers across countries and cultures. While there is valid concern that GPT may reinforce a WEIRD perspective (78, 79), GPT may be overall beneficial in moving computational social science beyond this WEIRD perspective, since it is better suited for multilingual analysis than prior computational social science tools. Overall, GPT might increase the ease and accessibility of advanced natural language processing methods, which may empower more researchers around the globe to do advanced text analysis research.

One limitation of our work is that we only compare GPT’s responses to the judgments of human annotators. We do not, however, show that GPT can accurately detect what a person is feeling or experiencing, though this may be of interest to future researchers. While the judgments of manual annotators are often considered the gold standard for validating natural language processing methods, this gold standard is still imperfect (72), and does not necessarily reflect the complex nature of the constructs we are measuring (80). Notably, there is often considerable disagreement between human annotators, which is also reflected in the datasets we analyzed (*SI Appendix*, Table S10). Because we did not have detailed demographic data on the annotators, it was difficult to examine whether GPT was biased toward reflecting the judgments of certain annotators. Additionally, while GPT strongly outperformed dictionary methods at detecting manually annotated sentiment and emotion, some of these dictionary methods, such as LIWC, were not necessarily designed to detect manually annotated sentiment. For instance, LIWC has been validated to be a measure that correlates with people’s behaviors or self-reports (31).

GPT also has other limitations that researchers may want to consider. First, the GPT API costs money to use, with GPT-4 being the most expensive. These price concerns might be especially pronounced for researchers in non-WEIRD contexts. However, this price of the GPT API is still much lower than other research costs—such as the cost of hiring human annotators to manually label data, or the cost of hiring experts to design novel machine learning classifiers. GPT’s API costs have reduced with successive model updates and will hopefully continue to do so.^†^ Despite this, researchers with fewer resources may still want to consider the many modern LLMs that are free or open-source (such as Large Language Model Meta AI, or LLaMA), which often approach the accuracy of GPT at many tasks (81). Finally, GPT uses text it receives for further training, raising important ethical considerations when using GPT to analyze private or sensitive data.

Finally, while GPT has been lauded as being one of the largest and most impressive language models, researchers may have reason to consider using other LLMs besides GPT (such as BERT, Bard, Claude, or LLaMA) for text analysis tasks and evaluate the strengths and weaknesses of each one (82). New LLMs are also being designed and released at a rapid pace, and future research should test the efficacy of future LLMs for text analysis tasks. Research should also explore different prompt variations, techniques, languages, and probe potential biases in more depth.

While new LLMs and other tools may eventually surpass GPT, we find that GPT is presently an accurate and easy to use text analysis tool that works across languages and contexts. During the revision of this paper, OpenAI released an improved GPT-4 model (GPT-4 Turbo) that was, at the same time, less expensive to use and more accurate, particularly in lesser-spoken languages. Even more recently, OpenAI released GPT-4o, which is cheaper and faster than GPT-4 Turbo.^‡^ We encourage researchers to evaluate new models for text analysis tasks as they continue to be released.

## Conclusions

Our results suggest that GPT is an effective tool for detecting various psychological constructs in text across several languages. GPT may have a number of benefits over existing text analysis methods, such as dictionary-based methods and fine-tuned machine learning models. It shows reasonable accuracy across languages and contexts, requires no training data, and is easy to use with little code and simple prompts. Therefore, we believe GPT and future LLMs may soon supplant existing automated text analysis approaches and facilitate more cross-linguistic research with lesser-resourced languages and non-WEIRD populations.

## Methods

### Datasets.

#### Selection of datasets and comparison models.

We selected as many publicly available datasets as possible to assess the generalizability of GPT. We aimed to select datasets that included a variety of texts (tweets, news headlines, and Reddit comments), languages (12 languages in total), and psychological constructs (sentiment, discrete emotions, offensiveness, and moral foundations) that were all evaluated by human annotators. With the exception of the news headlines dataset, these datasets came from prior studies that developed machine learning models for text analysis. Each study was the most recent analysis we could find for that particular construct-language pair, and for each study, we compared GPT to the top-performing model in that study. We took the model statistics for the top-performing model from the original papers, since it was often difficult for us to access the original model to rerun. In *SI Appendix*, Table S10, we provide details on annotators, interrater reliability, and preprocessing steps applied to these datasets.

#### Sentiment of English tweets.

We used the dataset of English tweets from SemEval-2017 Task 4: Sentiment Analysis on Twitter (67). Each tweet in this dataset was annotated by at least five human annotators from the crowdsourcing service CrowdFlower. We applied GPT to subtask A, which involved classifying the sentiment of each tweet into one of three classes: positive, negative, or neutral. We used the designated test set for subtask A (*N* = 12,284). Because of cost limitations, for GPT-4, we only analyzed the first 1,000 tweets.

#### Sentiment of Arabic tweets.

We also used the Arabic dataset from SemEval-2017 Task 4, which was similarly annotated using CrowdFlower. For consistency with the English sentiment analysis task, we chose subtask A for the Arabic data as well and tested the performance of GPT on the Arabic test set of subtask A (*N* = 6,100). Because of cost limitations, for GPT-4 and GPT-4 Turbo, we only analyzed the first 1,000 tweets.

#### Discrete emotions in English tweets.

To examine the performance of GPT at detecting discrete emotions in tweets, we applied it to a dataset from the TweetEval benchmark (83). This dataset was adapted from a previous one used in SemEval-2018 Task 1 (84), which was focused on emotion detection. The previous dataset contained tweets labeled with one or more of 12 emotion labels, following annotations by at least seven CrowdFlower workers for each tweet. The TweetEval dataset was created from this dataset by removing tweets with multiple labels and only keeping the four most common labels: anger, joy, sadness, and optimism. We used the test portion of this dataset (*N* = 1,421).

#### Discrete emotions in Indonesian tweets.

We used a dataset from the IndoNLU benchmark (85) to assess GPT’s performance on detecting discrete emotions in a different language from English. This was a dataset of tweets labeled with one of five emotions—anger, joy, sadness, fear, and love—by two annotators, taken from a previous study (86). We used the test portion of this dataset (*N* = 442).

#### Offensiveness in English tweets.

We used a dataset of English tweets from SemEval-2019 Task A: Offensive Language Identification (25). Each tweet was annotated by two people via the crowdsourcing platform Figure Eight. In the case of disagreement, a third annotator was used, and the annotation was decided by majority vote. Tweets were classified as either offensive or nonoffensive. We used the test dataset (*N* = 860).

#### Offensiveness in Turkish tweets.

We also used a dataset of Turkish tweets from SemEval-2020 Task 12: Multilingual Offensive Language Identification in Social Media (68). Most tweets were annotated by a single annotator. Tweets were classified as either offensive or nonoffensive. We used the test dataset (*N* = 3,528). Because of cost limitations, for GPT-4 and 4 Turbo, we only analyzed the first 1,000 tweets in the dataset.

#### Sentiment and discrete emotions in news headlines.

We used a dataset of 213 news headlines manually annotated for sentiment and discrete emotions (e.g., fear, joy, sadness, anger) (18). Manual annotations were made on a 1 to 7 scale by eight annotators, and averaged for each construct. This dataset was created to evaluate two common approaches for measuring sentiment and emotions in text: the NRC emotion lexicon (69) and the LIWC (34).

#### Sentiment analysis in African languages.

We analyzed a recent collection of datasets of tweets in various African languages. The tweets were manually coded for sentiment and used to develop multilingual machine learning models within one of the tasks at SemEval-2023–AfriSenti (70). Out of the 14 languages included, we excluded two Arabic dialects due to the overlap with our previous analysis of Arabic sentiment. We also excluded Mozambican Portuguese because it is a variety of Portuguese, meaning that GPT might perform better simply due to generalization from other varieties of Portuguese. Additionally, we excluded Nigerian Pidgin due to its lexical overlap with English, leading to the same potential generalization issue. Last, we excluded Tigrinya and Oromo, since the AfriSenti models were never trained on these languages (whereas GPT might have seen these languages in its training). For the remaining eight languages, we used their respective test sets. Due to cost constraints, we selected a random sample of 1,000 tweets for the datasets which had significantly more than 1,000 tweets.

#### Moral foundations in English-language Reddit posts.

Finally, we analyzed the Moral Foundations Reddit Corpus (64), a dataset of 16,123 Reddit comments that were manually annotated for specific moral foundations (i.e., i.e., Care, Proportionality, Equality, Purity, Authority, Loyalty) based on Moral Foundations Theory (87). We analyzed all comments in this dataset.

### GPT Procedure.

We used the OpenAI API to query GPT. The code for querying was written in R for GPT-3.5 and in Python for GPT-4 and GPT-4 Turbo. The GPT-4 and GPT-4 Turbo analysis was run-through the Microsoft Azure OpenAI API (with the exception of the GPT-4 Turbo analysis for news headlines, which was run-through the OpenAI API in R). Microsoft Azure sometimes triggered automatic content filters for sensitive topics. Anything that triggered a content filter warning as an output was filtered out. Analysis code was written in R. See https://osf.io/6pnb2/ for example code and data (66). We used a temperature of 0 to obtain the highest probability predictions of the models. This setting means that the GPT output would not largely differ if we ran our analysis a second time. Analyses were run in April 2023 for GPT-3.5 Turbo and GPT-4 for all datasets but the moral foundations dataset. Analyses with the moral foundations dataset with GPT-4 were run in January 2024. Analyses with GPT-4 Turbo were run in February 2024.

### GPT Prompts.

For each task, we used tailored prompts that included the relevant question followed by an instruction to provide the answer as a number and an explanation of what the numbers meant. The non-English versions were identical to the English versions, with the addition of the name of the respective language before the word “text” or “post.” The prompts were identical for the different runs. In most cases, we provided GPT with the exact same base prompts that annotators were provided, when these instructions were made available, with our custom prompt added asking GPT to answer only with a number. However, for the moral foundations prompts, we also told GPT the name of the moral foundation that they were annotating (in addition to just providing the definition, which the original annotators were told) after pretesting found that this slightly improved accuracy. Sample prompts are shown in Table 2.

### Text Preprocessing.

We did not apply any text preprocessing before GPT was used. We used the original datasets supplied by the authors. We report all preprocessing steps used by the authors of the papers whose datasets we used in *SI Appendix*, Table S10.

### Dictionary Analysis.

All dictionary analysis was conducted by Robertson et al. (18). Dictionary analysis was only used for dataset 7. Dictionary sentiment analysis was conducted using LIWC 2015. The positive and negative sentiment scores were calculated by finding the total number of positive words and the total number of negative words (as defined by the positive and negative sentiment dictionaries in LIWC 2015, negation handled) in a given headline, and dividing by the number of words in the headline overall. The single “sentiment” score was computed by taking the difference between the positive and negative scores for a given headline. For example, a headline that had a score of 0.3 for positive sentiment and 0.1 for negative sentiment would have a score of 0.2 for sentiment, while a headline that had a score of 0.2 for negative and 0 for positive would have a score of −0.2 for sentiment. We did not use separate dimensions for the statistical analysis with GPT.

For the discrete emotion analysis, we used the NRC dictionaries for Anger, Fear, Joy, and Sadness. We used only these four emotions because Robertson et al. (18) found that only those emotions were significantly correlated with human rater judgments. Human ratings were not significantly correlated with NRC codings for Trust, Surprise, Disgust, and Anticipation.

Dictionary scores also included negation handling. Specifically, any emotional word that followed within three words of a negation word was reverse coded. Thus, phrases like not happy, not too happy, or not looking too good were all coded as negative.

### Few-Shot Learning.

We ran GPT-4 with few-shot learning on each of the first 6 datasets to test its ability to improve performance over the default, zero-shot approach. To achieve few-shot learning, we added one example of text and its corresponding label taken from the same dataset to the prompt, which we then excluded from the analysis. An example prompt used for few-shot learning in the English discrete emotion detection task is shown as an example in *SI Appendix*, Table S3.

### Performance Evaluation Metrics.

We use a variety of different metrics to evaluate the performance of GPT. We keep the metrics that we use the same as the metrics reported in the papers from which our datasets originated so that we can compare the performance of GPT to other models. For binary or multilabel classification tasks, we use a number of metrics commonly used to evaluate machine learning classifiers, which are described in depth below. For the “continuous” (Likert scale) task, we look at Spearman’s correlations between GPT output and manual annotations.

#### Accuracy.

The classification accuracy was computed in each Twitter task by calculating the number of tweets which were identically coded by humans and GPT and dividing that number by the total number of tweets in the dataset. This simple metric has the issue that it is biased toward classes or labels with more data points (e.g., if a dataset has 90 positive tweets and 10 negative tweets, a classifier which labels all tweets as positive would have an accuracy of 90%).

#### Macroaveraged F1.

We used the macroaveraged *F1* score to quantify classification accuracy in a way that is less sensitive to imbalances in the datasets. The *F1* score of a classification model for a specific class (e.g., for detecting negative tweets vs. all other tweets) represents the harmonic mean of the model’s precision and recall.

$$F_{1}=2\frac{\mathrm{precision}\cdot \mathrm{recall}}{\mathrm{precision}+\mathrm{recall}}.$$

The precision represents the proportion of data points labeled with the given class by the classifier that are truly of that class (“true positives”) as opposed to falsely labeled (“false positives”). In the negative tweets example, precision would be the proportion of tweets labeled as negative by the classifier that are actually negative.

$$\mathrm{precision}=\frac{\mathrm{true}\;\mathrm{positives}}{\mathrm{true}\;\mathrm{positives}+\mathrm{false}\;\mathrm{positives}}.$$

The recall represents the ratio of true positives over the sum of true positives and false negatives (members of the class which are wrongly labeled by the classifier as not belonging to the class). In our example, recall is the proportion of tweets that are actually negative that are labeled by the classifier as negative.

$$\mathrm{recall}=\frac{\mathrm{true}\;\mathrm{positives}}{\mathrm{true}\;\mathrm{positives}+\mathrm{false}\;\mathrm{negatives}}.$$

In each Twitter task, the *F1* score for each class was calculated (e.g., for negative tweets vs. all others, for positive tweets vs. all others, etc.) and the arithmetic mean of all *F1* scores was computed to give the macroaveraged *F1*. In the SemEval-2017 datasets, following the methodology of the initial study, we computed the macroaveraged *F1* score only for the positive and negative classes.

#### Spearman correlation.

The results in the news headline task, which was coded on a 1 to 7 Likert scale, were evaluated by Spearman correlation between the GPT and human values for the different constructs (sentiment and the four basic emotions).

## Supplementary Material

Appendix 01 (PDF)

## Data Availability

Data and code data have been deposited in Open Science Framework (OSF) (https://osf.io/6pnb2/) (66).

## Footnotes

^*^This dataset was posted on our OSF in April 2023 as part of the OSF for the initial pre-print of this paper. This means that GPT-4 Turbo, which has training data up to December 2023, might have had access to this dataset. However, given that GPT-4—which only had data access up to April 2023—had very similar performance to GPT-4 Turbo, we do not think that data contamination is a major issue.

^†^OpenAI announced reduced prices with the announcement of the updated GPT-4 Turbo model on 25 January 2024.

^‡^See more information about GPT-4o here: https://platform.openai.com/docs/models. We have not yet evaluated the performance of GPT-4o.
