# Supplementary material for: GPT is an effective tool for multilingual psychological text analysis
Source: Proc Natl Acad Sci U S A. 2024 Aug 12;121(34):e2308950121. doi: 10.1073/pnas.2308950121 (PMC11348013; doi:10.1073/pnas.2308950121)
Supplement: Supplementary file 1 — Appendix 01 (PDF) [file pnas.2308950121.sapp.pdf]

Supplementary Appendix for:

GPT is an effective tool for multilingual psychological text analysis

Steve Rathje[1]*, Dan-Mircea Mirea[2]*, Ilia Sucholutsky[3], Raja Marjieh[2], Claire E. Robertson[1], Jay J. Van Bavel[1]

*these authors contributed equally

Supplementary Table S1. Precision and recall values for the different versions of GPT on the sentiment, discrete emotions and offensiveness datasets

		**GPT-3.5 Turbo**		**GPT-4**		**GPT-4 Turbo**	
**Language**	**Construct**	**Precision**	**Recall**	**Precision**	**Recall**	**Precision**	**Recall**
English	Sentiment	0.639	0.700	0.608	0.711	0.543	0.717
Arabic	Sentiment	0.701	0.695	0.677	0.690	0.700	0.731
English	Discrete emotions	0.710	0.745	0.772	0.789	0.777	0.797
Indonesian	Discrete emotions	0.753	0.675	0.783	0.734	0.789	0.788
English	Offensiveness	0.718	0.725	0.754	0.740	0.720	0.730
Turkish	Offensiveness	0.756	0.748	0.816	0.673	0.848	0.722
Swahili	Sentiment	0.543	0.615	0.488	0.614	0.507	0.642
Hausa	Sentiment	0.652	0.590	0.614	0.450	0.742	0.689
Amharic	Sentiment	0.411	0.386	0.620	0.606	0.682	0.623
Yoruba	Sentiment	0.604	0.522	0.671	0.583	0.707	0.715
Igbo	Sentiment	0.653	0.581	0.677	0.603	0.622	0.654
Twi	Sentiment	0.528	0.471	0.522	0.517	0.499	0.500
Kinyarwanda	Sentiment	0.582	0.573	0.627	0.623	0.699	0.693
Tsonga	Sentiment	0.625	0.432	0.562	0.443	0.540	0.510
**Average**	-	**0.634**	**0.604**	**0.657**	**0.627**	**0.670**	**0.679**

Values are averages across all categories (e.g. for sentiment, precision and recall were calculated for each of ‘positive’,’neutral’ and ‘negative’ labels, and the average across these labels was computed). Because of cost limitations, we only ran GPT-4 and 4 Turbo only on the first 1000 rows of the datasets that significantly exceeded 1000 tweets.

Supplementary Table S2. GPT-4 Zero-Shot vs. Few-Shot Learning (prompt with one example per class).

**Model**	**Zero-shot**			**Few-shot**		
**Metric**	**Accuracy**	**F1**	**Recall**	**Accuracy**	**F1**	**Recall**
Sentiment of English tweets (2017)	0.566	0.633	0.711	0.654	0.647	0.704
Sentiment of Arabic tweets (2017)	0.655	0.707	0.690	0.665	0.696	0.687
Discrete emotions in English tweets (2020)	0.816	0.779	0.789	0.808	0.775	0.803
Discrete emotions in Indonesian tweets (2020)	0.741	0.740	0.734	0.782	0.789	0.778
Offensiveness in English tweets (2019)	0.801	0.746	0.740	0.796	0.677	0.656
Offensiveness in Turkish tweets (2020)	0.857	0.709	0.673	0.859	0.691	0.652

We examine the improvement in GPT's performance through "few-shot learning," accomplished by providing an example of each class within the prompt. For example, for the discrete emotions task, we provided GPT with one example of each discrete emotion followed by the emotion label. Few-shot learning improved performance in some cases but hurt performance in other cases. Green indicates instances in which few-shot learning showed improvement over zero-shot learning.

Supplementary Table S3. Prompt used for few-shot learning in English emotion detection.

Which of these four emotions - anger, joy, sadness, optimism - best represents the mental state of the person writing the following text? Answer only with a number: 1 if anger, 2 if joy, 3 if sadness, 4 if optimism. Here are some examples:

Text: #Deppression is real. Partners w/ #depressed people truly dont understand the depth in which they affect us. Add in #anxiety &makes it worse
Emotion: 3

Text: [USER] Welcome to #MPSVT! We are delighted to have you! #grateful #MPSVT #relationships
Emotion: 2

Text: sleep is and will always be one of the best remedies for a tired and weary soul
Emotion: 4

Text: [USER] how can u have sold so many copies but ur game has so many fucking bugs and mad lag issues. Optimize ur shit soon.
Emotion: 1

Here is the text: [Tweet]
Emotion:

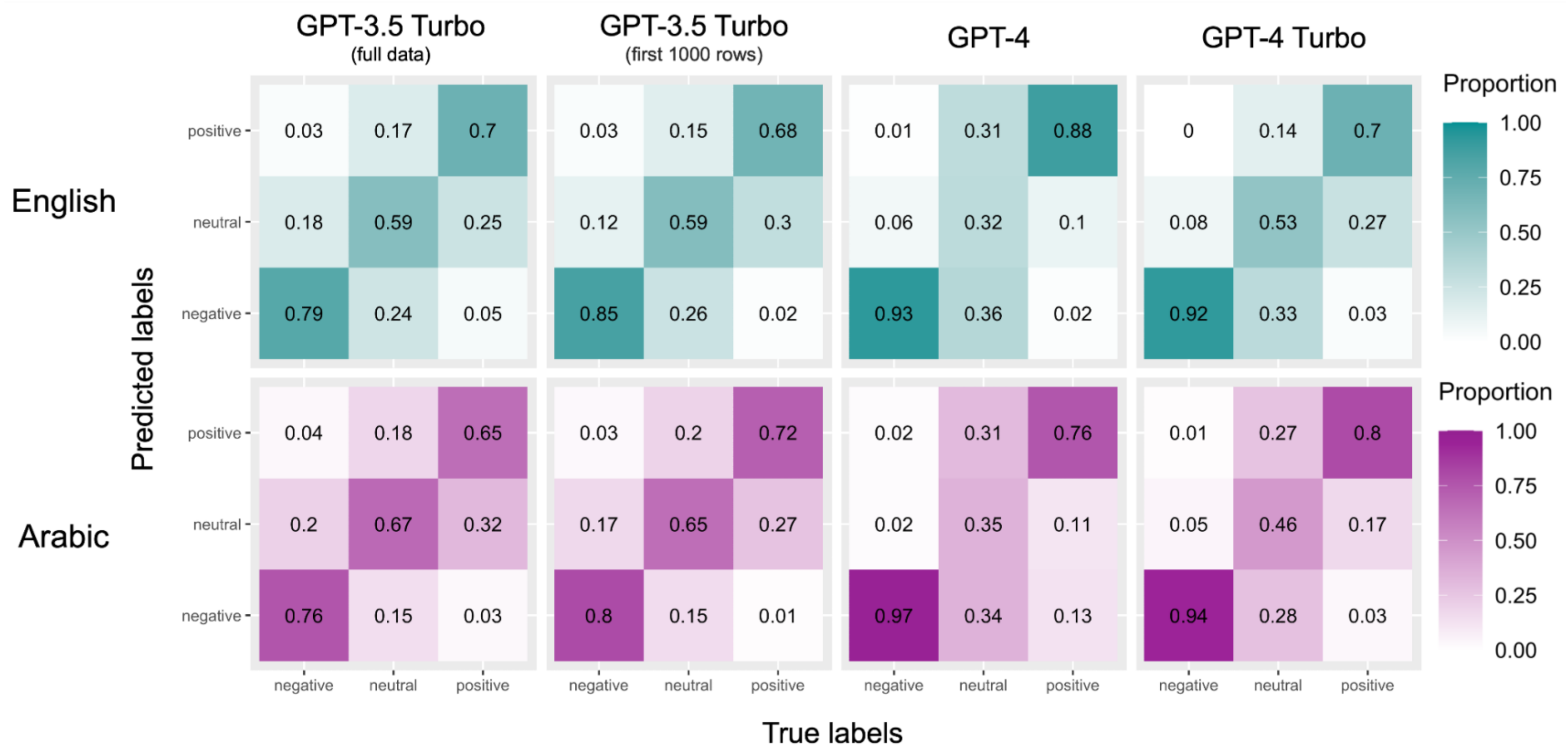

Supplementary Figure S1. Confusion matrices of GPT-labeled ('predicted') sentiment vs. human-labeled ('true') sentiment. Numbers show the proportion of tweets labeled by humans with a particular label that received a particular GPT label (numbers sum up to 1 in each column). Darker colors reflect higher proportions. Colors reflect language: turquoise for English and violet for Arabic. The first two columns represent predictions made by GPT-3.5 Turbo on the full dataset (left-most column) and the first 1000 rows (which was added to facilitate comparison with GPT-4 and 4 Turbo). The last two columns represent predictions made by GPT-4 and GPT-4 Turbo. Due to cost limitations, only the first 1000 rows were run for these models. As shown, GPT-4 appears to have a bias toward labeling neutral tweets as positive or negative, which persists in GPT-4 Turbo to a lesser extent.

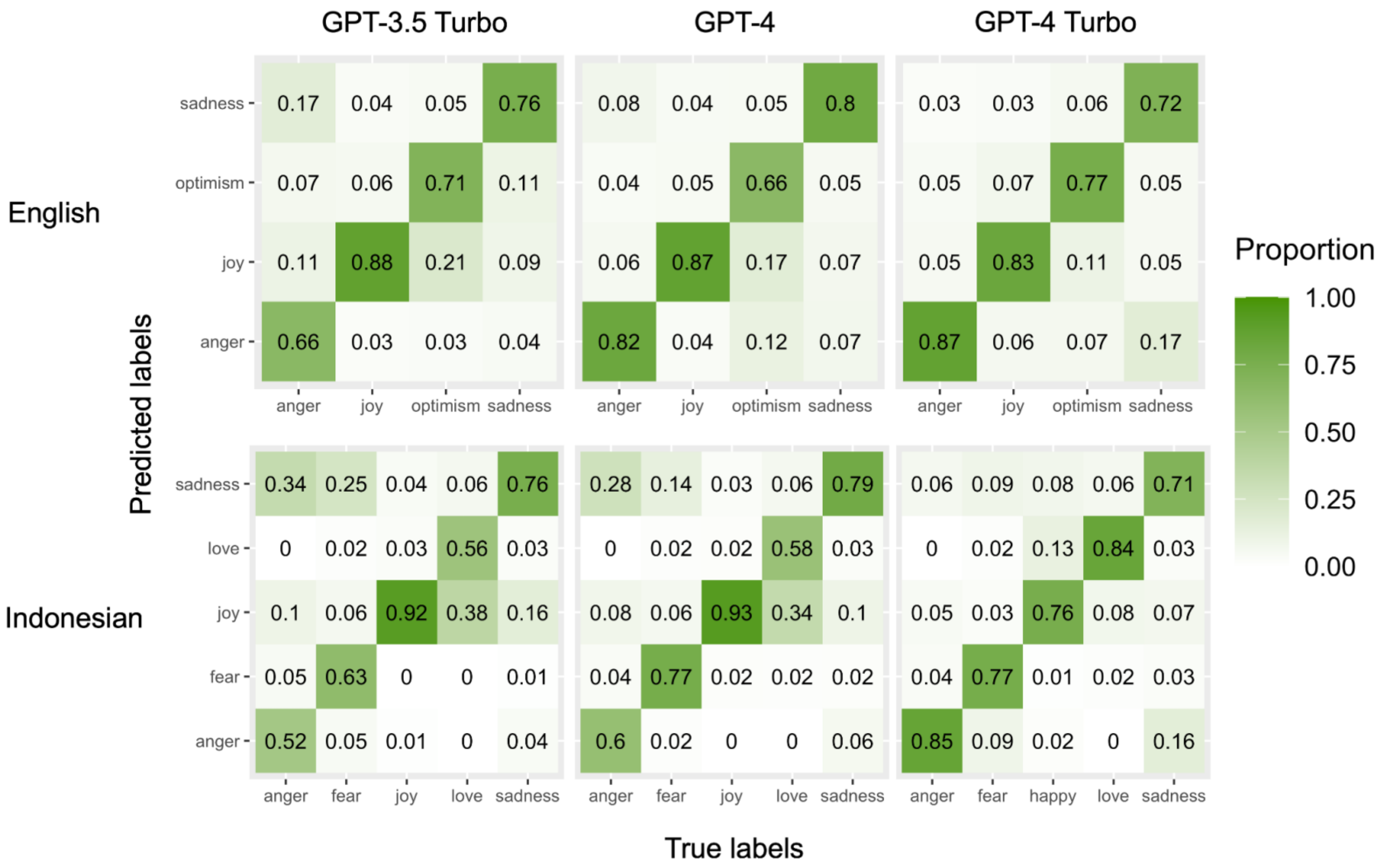

Supplementary Figure S2. Confusion matrices for discrete emotion detection. Numbers show the proportion of tweets labeled by humans with a particular label (on the X-axis) that received a particular GPT label (on the Y-axis; numbers sum up to 1 in each column). Darker colors reflect higher proportions. Left heatmaps: GPT-3.5 Turbo predictions; center heatmaps: GPT-4 predictions; right heatmaps: GPT-4 Turbo predictions; top heatmaps: results for English tweets; bottom heatmaps: results for Indonesian tweets. As shown, performance increased with each version of GPT, as indicated by larger numbers and darker colors along the diagonal. In particular, the confusion matrices suggest GPT got better at detecting anger and became less likely to label a tweet as expressing joy (leading to less true positives but also less false positives).

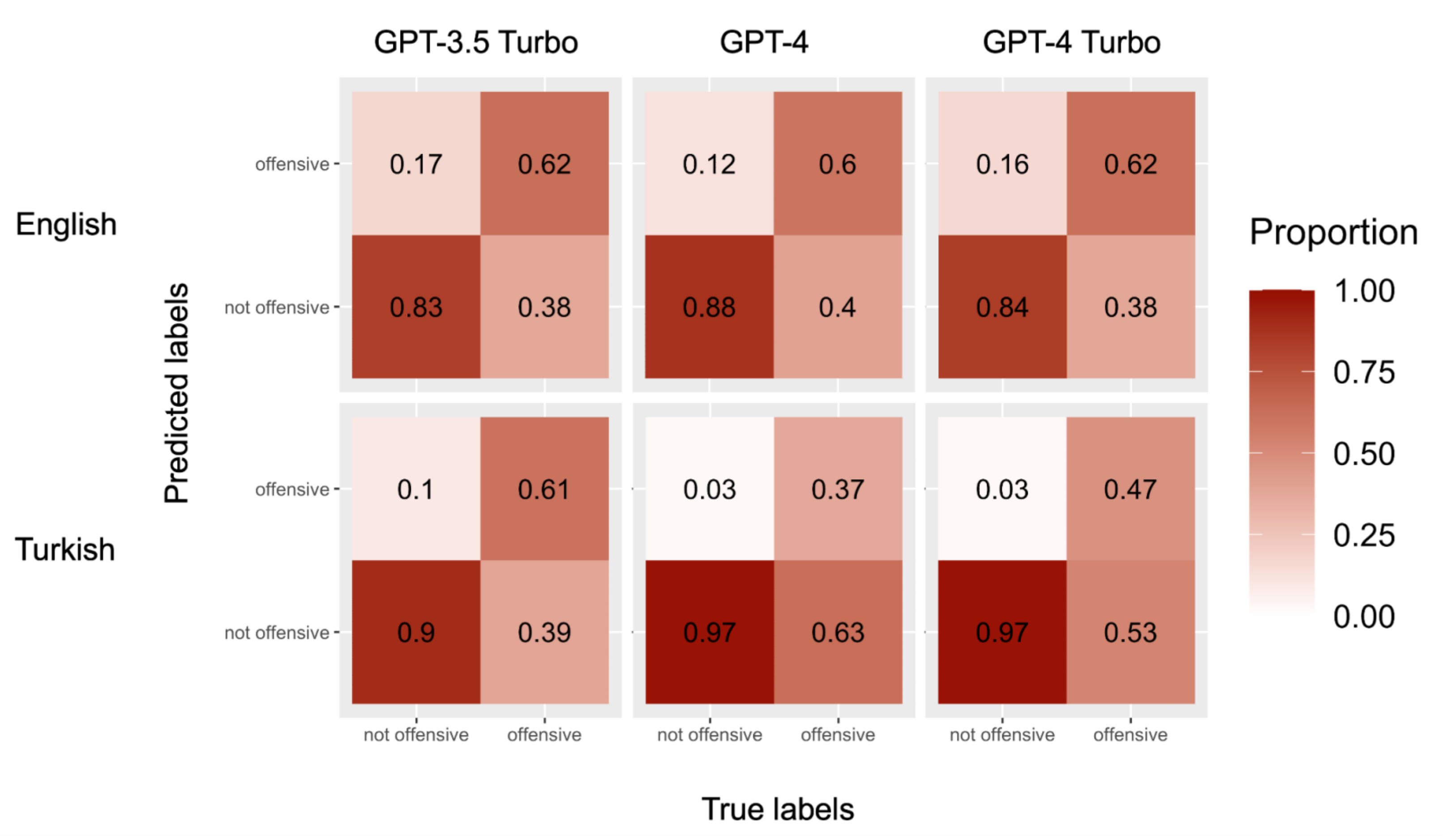

Supplementary Figure S3. Confusion matrices for offensiveness detection. Numbers show the proportion of tweets labeled by humans as either ‘offensive’ or ‘not offensive’ (on the X-axis) that received a particular GPT label (on the Y-axis; numbers sum up to 1 in each column). Darker colors reflect higher proportions. Left heatmaps: GPT-3.5 Turbo predictions; center heatmaps: GPT-4 predictions; right heatmaps: GPT-4 Turbo predictions; top heatmaps: results for English tweets; bottom heatmaps: results for Turkish tweets. Performance is similar between the different versions for the English dataset. For the Turkish dataset, performance decreased from GPT-3.5 Turbo to GPT-4 due a lower tendency of labeling tweets as offensive. This tendency was ameliorated in GPT-4 Turbo.

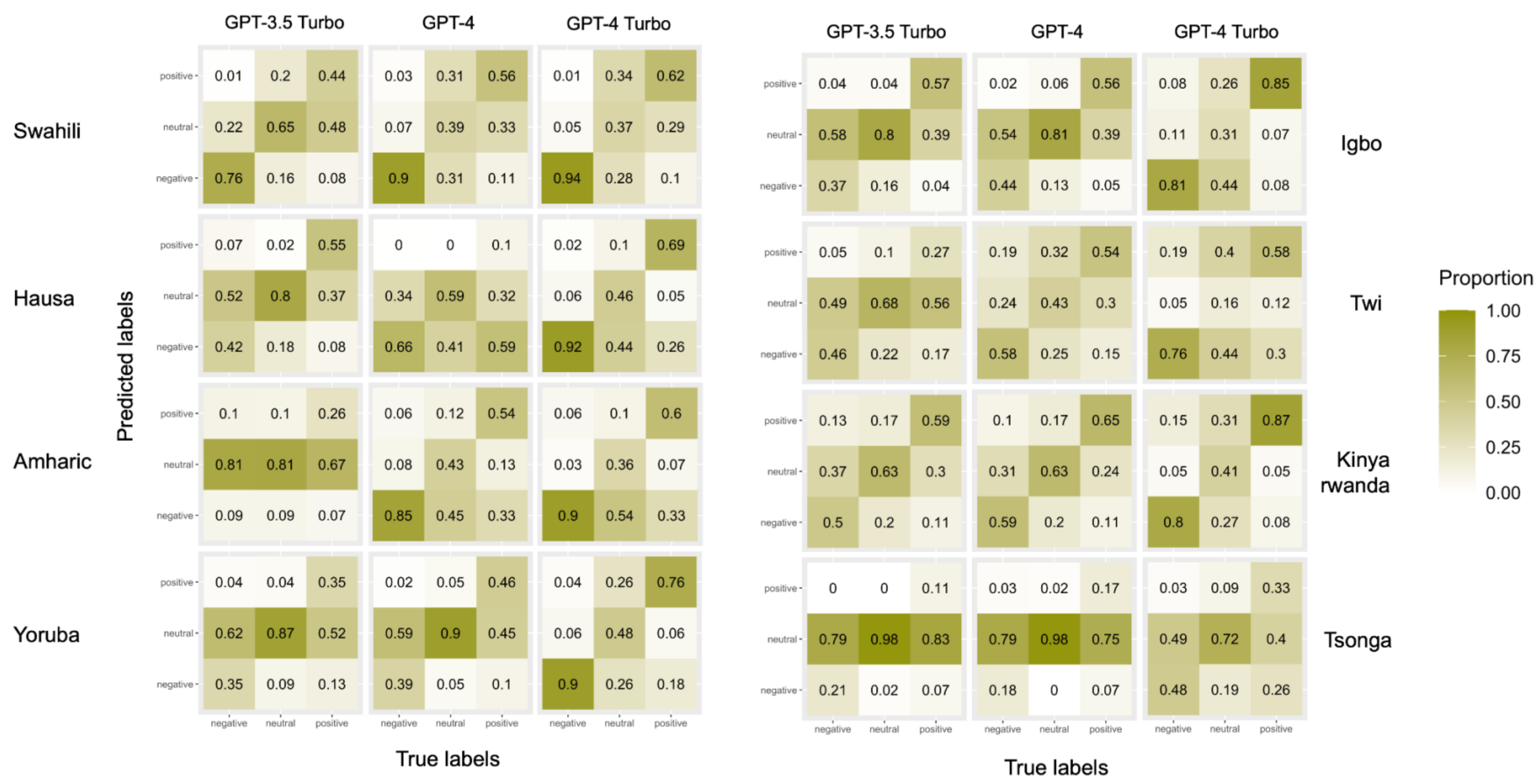

Supplementary Figure S4. Confusion matrices for sentiment detection in the African languages. Numbers show the proportion of tweets labeled by humans with a particular label (on the X-axis) that received a particular GPT label (on the Y-axis; numbers sum up to 1 in each column). Darker colors reflect higher proportions. Left heatmaps: GPT-3.5 Turbo predictions; center heatmaps: GPT-4 predictions; right heatmaps: GPT-4 Turbo predictions; top heatmaps: results for English tweets; heatmaps for different languages are stacked on top of each other and on top columns. Performance generally improved with each successive GPT version, as indicated by darker colors along the diagonal. At the same time, newer versions showcase an 'extremity bias' across languages, being less likely to label tweets as 'neutral'. This is signaled by lighter colors along the middle rows (corresponding to neutral predictions) and darker colors along the top and bottom rows (corresponding to positive and negative predictions, respectively).

Supplementary Table S4: Correlations between dictionary methods and GPT output

	**Spearman Correlation between GPT-3 results and dictionary methods**	**Spearman Correlation between GPT-4 results and dictionary methods**	**Spearman Correlation between GPT-4 Turbo results and dictionary methods**
**Sentiment**	0.27	0.38	0.35
**Anger**	0.16	0.22	0.22
**Fear**	0.32	0.34	0.32
**Joy**	0.28	0.26	0.12
**Sadness**	0.19	0.27	0.23

Supplementary Table S5: Moral foundations task - comparison of GPT-4 with a fine-tuned BERT Model

Psychological Construct	GPT-4				Fine-Tuned BERT		
	Accuracy	F1	Precision	Recall	F1	Precision	Recall
Care	0.90	0.53	0.63	0.47	0.62	0.58	0.66
Equality	0.91	0.36	0.47	0.29	0.58	0.61	0.56
Proportionality	0.94	0.17	0.56	0.10	0.37	0.44	0.33
Loyalty	0.95	0.34	0.39	0.30	0.45	0.48	0.41
Authority	0.93	0.21	0.38	0.15	0.40	0.39	0.41
Purity	0.98	0.14	0.32	0.19	0.51	0.69	0.42
Moral Sentiment	0.63	0.65	0.49	0.94	0.73	0.74	0.73

GPT-4 output compared to output from a fine-tuned BERT model from the original Moral Foundations Reddit Corpus data (Trager et al., 2022).

Supplementary Table S6: Moral foundations task - comparison of GPT-4 Turbo with a fine-tuned BERT Model

Psychological Construct	GPT-4 Turbo				Fine-Tuned BERT		
	Accuracy	F1	Precision	Recall	F1	Precision	Recall
Care	0.90	0.50	0.65	0.40	0.62	0.58	0.66
Equality	0.92	0.24	0.54	0.16	0.58	0.61	0.56
Proportionality	0.94	0.13	0.58	0.07	0.37	0.44	0.33
Loyalty	0.96	0.26	0.41	0.19	0.45	0.48	0.41
Authority	0.93	0.09	0.38	0.05	0.40	0.39	0.41
Purity	0.98	0.14	0.40	0.09	0.51	0.69	0.42
Moral Sentiment	0.68	0.68	0.54	0.92	0.73	0.74	0.73

GPT-4 Turbo output compared to output from a fine-tuned BERT model from the original Moral Foundations Reddit Corpus data (Trager et al., 2022).

Supplementary Table S7: Moral Foundations Compared to an alternate fine-tuned BERT model trained on Twitter data

Psychological Construct	GPT-4 F1	GPT-4 Precision	GPT-4 Recall	Fine-Tuned BERT F1 (Trained On Twitter Data)	Fine-Tuned BERT Precision (Trained On Twitter Data)	Fined-Tuned BERT Recall (Trained On Twitter Data)	Year of Study
Care	0.53	0.63	0.47	0.43	0.53	0.35	2022
Loyalty	0.34	0.39	0.30	0.32	0.48	0.23	
Authority	0.21	0.38	0.15	0.31	0.23	0.44	
Purity	0.14	0.32	0.19	0.34	0.52	0.25	

GPT-4 performs worse than a fine-tuned BERT model that was trained on Twitter (and not Reddit) data for the foundations of Care and Loyalty. The fine-tuned BERT model was reported in the original Moral Foundations Reddit Corpus paper (Trager et al., 2022).

Supplementary Table S8: Moral Foundations Compared to an alternate fine-tuned BERT model trained on Twitter data

Psychological Construct	GPT-4 F1	GPT-4 Precision	GPT-4 Recall	Fine-Tuned BERT F1 (Trained On Twitter Data)	Fine-Tuned BERT Precision (Trained On Twitter Data)	Fined-Tuned BERT Recall (Trained On Twitter Data)	Year of Study
Care	0.50	0.65	0.40	0.43	0.53	0.35	2022
Loyalty	0.26	0.41	0.19	0.32	0.48	0.23	
Authority	0.09	0.38	0.05	0.31	0.23	0.44	
Purity	0.14	0.40	0.09	0.34	0.52	0.25	

GPT-4 Turbo performs worse than a fine-tuned BERT model that was trained on Twitter (and not Reddit) data for the foundations of Care. The fine-tuned BERT model was reported in the original Moral Foundations Reddit Corpus paper (Trager et al., 2022).

Supplementary Table S9. GPT-4 Turbo test-retest reliability

**Language**	**Construct**	**Weighted Cohen's κ**
Indonesian	Discrete emotions	0.95*
Swahili	Sentiment	0.99
Hausa		0.99
Amharic		0.95
Yoruba		0.99
Igbo		0.97
Twi		0.98
Kinyarwanda		0.99
Tsonga		0.93

Test-retest reliability values (measured using weighted Cohen's kappa) of GPT-4 Turbo. The test and the retest were one day apart from the African languages and simultaneous for Indonesian. *For Indonesian, the retest was performed with the prompt in the original language (Indonesian). The translation was performed via Google translate.

Supplementary Table S10. Information about annotators, reliability, and pre-processing across all datasets

**Dataset**	**Construct**	**Language**	**Information About Annotators**	**Reliability Statistic Reported**	**Reliability**	**Text Preprocessing**
Sentiment of English tweets (2017)	Sentiment	English	Each tweet was annotated by at least 5 people taken from the crowdsourcing site CrowdFlower	NA	NA	No text preprocessing steps are described
Sentiment of Arabic tweets (2017)	Sentiment	Arabic	Each tweet was annotated by at least 5 people taken from the crowdsourcing site CrowdFlower	NA	NA	No text preprocessing steps are described
Discrete emotions in English tweets (2020)	Discrete Emotions	English	Tweets were annotated via the crowdsourcing site CrowdFlower. 303 people annotated between 10 and 4,670 tweets each. The median number of annotators people tweet was 7.	Inter-Rater Agreement/Fleiss's K	0.81/0.40	No text preprocessing steps are described.
Discrete emotions in Indonesian tweets (2020)	Discrete Emotions	Indonesian	Each tweet was annotated by two people (no demographic data provided).	Agreement/Kappa Score	0.92/0.76	Sentences are separated by a new line. Private information in Twitter text is masked, so it appears like <username>, <hashtag>, <email>, and <link>.
Offensiveness in English tweets (2019)	Offensiveness	English	Each tweet was annotated by two people. Only "experienced" annotators were used on the platform Figure Eight. In case of disagreement, a third annotator was used	NA	NA	Several teams in this SemEval competition indicated that they used pre-processing techniques, which included normalizing tokens, hashtags, retweets, and URLs, converting emojis to text, or using tokenizers. It was not specified what teams used these pre-processing techniques.
Offensiveness in Turkish tweets (2020)	Offensiveness	Turkish	Annotators were native Turkish speakers recruited via the author's personal contacts.	Cohen's K	0.76	Text was converted to lowercase, punctuation was removed, and symbols and hashtags were removed. The

						tweets were then tokenized by the NLTK tokenizer.
Sentiment & discrete emotions in news headlines (2023)	Sentiment	English	8 NYU undergraduate students from the student subject pool	Kendall's W	0.33	The text was converted into lower-case and tokenized. Special characters (e.g., punctuation, hashtags, etc.) were removed.
	Anger	English			0.24	
	Fear	English			0.23	
	Joy	English			0.15	
	Sadness	English			0.23	
Sentiment of African tweets (2023)	Sentiment	Swahili	Each tweet was manually annotated by at least three native speakers, with annotation procedures differing slightly between languages. No demographic data was provided on the annotators.	Free Marginal Multirater Kappa	-	The authors anonymized all tweets by replacing all @mentions by @user and removing all URLs. For the Nigerian, tweets were lower-cased as well.
	Sentiment	Hausa			0.66	
	Sentiment	Amharic			0.47	
	Sentiment	Yoruba			0.65	
	Sentiment	Igbo			0.61	
	Sentiment	Twi			0.51	
	Sentiment	Kinyarwanda			0.43	
	Sentiment	Tsonga			0.50	
Moral Sentiment in Reddit Comments (2022)	Care	English	A pool of 27 annotators, who were all undergraduate research assistants at the University of Southern California, were selected. These annotators went through two months of training about moral foundations. The top five annotators were selected to perform final annotations. Each Reddit post was annotated by at least three annotators.	Fleiss's Kappa	Scores are shown graphically in the original paper using a heatmap (not using actual numbers). Thus, Kappa scores cannot be reported here.	No text preprocessing steps were described
	Equality	English				
	Proportionality	English				
	Loyalty	English				
	Authority	English				
	Purity	English				
	Moral Sentiment	English				

The above table contains information about the annotators of each dataset, the reliability statistics reported in the original paper about annotator agreement, and any text pre-processing steps applied to the dataset.

Supplementary References

1. Trager, J., Ziabari, A. S., Davani, A. M., Golazizian, P., Karimi-Malekabadi, F., Omrani, A., ... & Dehghani, M. (2022). The moral foundations reddit corpus. *arXiv preprint arXiv:2208.05545*.
